# RepARK—*de novo* creation of repeat libraries from whole-genome NGS reads

**DOI:** 10.1093/nar/gku210

**Published:** 2014-03-14

**Authors:** Philipp Koch, Matthias Platzer, Bryan R. Downie

**Affiliations:** Genome Analysis, Leibniz Institute for Age Research - Fritz Lipmann Institute, Beutenbergstr. 11, 07745 Jena, Germany

## Abstract

Generation of repeat libraries is a critical step for analysis of complex genomes. In the era of next-generation sequencing (NGS), such libraries are usually produced using a whole-genome shotgun (WGS) derived reference sequence whose completeness greatly influences the quality of derived repeat libraries. We describe here a *de novo* repeat assembly method—RepARK (Repetitive motif detection by Assembly of Repetitive K-mers)—which avoids potential biases by using abundant k-mers of NGS WGS reads without requiring a reference genome. For validation, repeat consensuses derived from simulated and real *Drosophila melanogaster* NGS WGS reads were compared to repeat libraries generated by four established methods. RepARK is orders of magnitude faster than the other methods and generates libraries that are: (i) composed almost entirely of repetitive motifs, (ii) more comprehensive and (iii) almost completely annotated by TEclass. Additionally, we show that the RepARK method is applicable to complex genomes like human and can even serve as a diagnostic tool to identify repetitive sequences contaminating NGS datasets.

## INTRODUCTION

Repetitive DNA is widespread among eukaryotes and generation of accurate repeat libraries is critical for genomic analyses: >50% of the human genome is composed of repeats (1), while some important agricultural crops such as barley have more than 80% repetitive sequence (2). In many sequence and genome analyses such as read alignment, *de novo* genome assembly and genome annotation, repeats can present major challenges (3). Identification and classification of repeats is one of the first steps in genome annotation as transposons can contain features such as protein-coding regions that complicate subsequent analyses (e.g. gene annotation) if repeats are not properly marked (4). Additionally, repeats are believed to play significant roles in genome evolution (5) and disease (6–7).

Depending on their size and distribution, repetitive elements are categorized into different types. Tandem repeats are composed of highly conserved sequence motifs located directly adjacent to each other, have unit sizes from 1 to more than 100 bp, and are categorized into microsatellites, minisatellites or satellites based on their unit size (8). Dispersed repeats range between 50 bp and 30 kb, but are scattered throughout the entire genome (9). Segmental duplications (SDs) are low-copy repetitive regions of between 1 kb and several Mb in size with an identity ≥90%, and can occur either intra- or interchromosomally (10).

In the genomics era, repeat libraries are usually derived from a draft genome sequence. Following genome assembly, low complexity repeats such as tandem repeats are first predicted with Tandem Repeats Finder (11). RepeatMasker (http://www.repeatmasker.org) identifies and masks dispersed repeats using consensuses from RepBase Update (12), which contains manually curated repeat consensuses from hundreds of species. Both false positives (due to sequence similarities) and negatives (when repeats are highly divergent) can emerge at this stage. Species-specific repeat families can be identified *ab initio* from reference genomes using RECON (13), which evaluates pair-wise similarities to build repeat consensuses, or RepeatScout (14) which identifies and uses highly frequent k-mers as seeds that are extended based on multiple sequence alignments. Both of these programs rely on either a high-quality reference sequence or long Sanger-length sequencing reads. REPuter (15) and Repseek (16) both adopt a seed-and-extend paradigm to identify identical and degenerate repetitive sequence. P-clouds (17) determines repetitive motifs by clustering similar but divergent sequences together. ReAS (18) generates repeat libraries based on identification and extension of seeds directly from shotgun reads rather than assembled sequences, but is limited to reads larger than 100 bp (the seed size) and has seen only limited usage (e.g. Drosophila 12 genomes project (19)). Tallymer predicts repeats based on k-mer counting in reference genomes and has identified repeats in the maize genome (20), but also relies on Sanger-length reads. Moreover, ‘surrogates’ generated as side-product when running ‘wgs-assembler’ (also known as Celera assembler) (21) represent sequences predicted to be repetitive based on depth of coverage statistics. Bambus 2, a scaffolder specifically adopted to metagenome datasets, can identify ‘variant motifs’ independent of coverage (22). Graph-based variation detection tools such as Cortex (23) can also be used to *de novo* identify genomic repeats, but require multiple samples or a finished reference genome. Finally, SDs may be detected via genome-wide all-versus-all alignments that are filtered to fulfill the requirements of ≥1 kb size and ≥90% identity (24). DupMasker uses information from a pre-defined SD-library to automatically detect SDs, but the SD-library limits this application to the human and other primate genomes (25). To date, there exist no resources to identify short length (<1 kb) or more divergent (<90% identity) SD events.

New opportunities in genome analysis have emerged with the advent of high-throughput short-read next-generation sequencing (NGS) technologies (3). However, complex, repeat-rich genomes still present major challenges for modern *de novo* assembly algorithms such as EULER (26), Velvet (27), ABySS (28), SOAPdenovo (29), ALLPATHS-LG (30) and CLC Assembly Cell (CLCbio, http://www.clc-bio.com). In the de Bruijn graph paradigm that dominates assembly algorithms for such genomes, reads are broken into sub-strings of k nucleotides (k-mers) and used to construct a directed graph. A genome assembly is derived from a path through this graph and repetitive genomic sequences lead to ambiguities while traversing the graph (3) and introduce structural assembly errors such as chimeric or mis-assembled contigs. In general, highly repetitive genomes usually lead to fragmented genome assemblies with an underrepresentation of repetitive content in the final assembly (31), but can also lead to false assembly repeats in the form of SDs (32–33).

To address these challenges, k-mer analysis is an important first step in most genome assembly projects. At this stage, k-mers of NGS reads are counted and plotted on a histogram. Such a histogram can be used to predict sequencing errors (34), genome size (35) or repetitive sequences in reads for purposes such as repeat content assessment (20) or scaffolding and gap filling (B. R. Downie, P. Koch, N. Jahn, J. Schumacher and M. Platzer, unpublished results). K-mers derived from the unique fraction of the genome will accumulate in a Poisson-like curve with a peak near the genome coverage, while sequences that occur more than once genome wide are progressively enriched among k-mers with higher coverages.

We postulated that de Bruijn graph assemblers could create a repeat library using only ‘abundant’ k-mers (those k-mers that are predicted to occur more than once genome wide). As a proof of principle, we used both simulated and real NGS data from the *Drosophila melanogaster* genome to create, validate and annotate *de novo* repeat libraries. Velvet, a widely used de Bruijn graph-based *de novo* genome assembler, assembled the NGS sequences from which RepeatScout predicted repeat consensuses, and wgs-assembler surrogates were extracted after a *de novo* genome assembly of the same NGS data. These repeat libraries were compared to that of RepBase update and to the ReAS *de novo* repeat library (ReASLib) from the Drosophila 12 genomes project (19). Finally, we validated 'Repetitive motif detection by Assembly of Repetitive K-mers' (RepARK) on a human Illumina DNA dataset produced for the ALLPATHS-LG publication (30) to ensure its applicability to larger, more complex genomes.

## MATERIALS AND METHODS

### The *Drosophila melanogaster* genome

The *D. melanogaster* R5.43 assembly (170 Mb) is distributed across 15 sequence entries: the left and right arms of chromosomes 2 and 3, chromosome X, the corresponding heterochromatin content of these chromosomes, chromosome Y only as heterochromatin, the mini chromosome 4, the mitochondrial genome, and 40 Mb in two additional pseudo-chromosomes (U and Uextra). Currently, 412 repeat consensuses in RepBase Update (release 20120418) can be extracted with the term ‘*drosophila melanogaster*’, of which 249 are non-low-complexity repeats including 26 that are *D. melanogaster*-specific repeats (i.e. non-ancestral). We also downloaded the *D. melanogaster* repeat library created in the 12 *Drosophila* genomes project using ReAS (ftp://ftp.genomics.org.cn/pub/ReAS/drosophila/v2/consensus_fasta/dmel.con.fa.gz) (391 consensuses).

### Sequencing data

Sixty-eight million 101 bp reads (‘simulated’; 27 average quality, 40× genome coverage, insert sizes 400 bp and 2500 bp) were simulated with MAQ (version 0.7.1, http://maq.sourceforge.net) without mutations or indels using an Illumina training dataset on the *D. melanogaster* genome release R5.43 (including the U and Uextra chromosomes). Additionally, two sets of experimentally obtained Illumina reads (‘real’; ycnbwsp_2: SRX040484; ycnbwsp_7-HE: SRX040486; 83 million reads, 82 nt avg. length, 30 average quality, 40× genome coverage) were downloaded from the Short Read Archive (http://www.ncbi.nlm.nih.gov/sra). They are derived from an individual of the stock (http://flybase.org/reports/FBst0002057.html) that was used in the release 5 of the *D. melanogaster* genome assembly (36). Both simulated and real datasets were error-corrected with QUAKE (34) (version 0.3.4, using default settings and *k* = 17). Human Illumina reads derived from a lymphoblastoid cell line (Coriell Institute, GM12878) (101 bp length, 132 Gb total, ∼40× coverage) were downloaded from SRA (SRR067780, SRR067784, SRR067785, SRR067787, SRR067789, SRR067791, SRR067792 and SRR067793) and used directly without error correction.

### Building the RepARK repeat libraries

For NGS *de novo* repeat library creation, k-mers of NGS whole-genome shotgun (WGS) datasets were first counted with Jellyfish (37) (version 1.1.6) using the highest supported k-mer size of 31 (−m 31, −both-strands). The threshold for ‘abundant’ k-mers (those occurring more than once genome wide) was predicted for each dataset. A histogram of k-mer frequencies is calculated and a linear function is fit to the slope of the descending segment of the Poisson-like unique k-mer fraction. k-mers which occur with a frequency above which the projected linear function crosses the x-axis are expected to occur more than once genome wide. To further ensure that no contamination of the abundant k-mer set by unique sequences occurred, this value was doubled, and k-mers with a frequency above this threshold were classified as abundant (simulated: k-mer coverage >60, real: >84, human: >76; Supplementary Figure S1). Abundant k-mers were isolated and independently *de novo* assembled using CLC Assembly Cell (CLC) (version 4.0) or Velvet (version 1.2.08) with default settings and k-mer size of 29, resulting in four RepARK *de novo* repeat libraries.

Additionally, repeat libraries for both real and simulated datasets were *de novo* generated using two established methods. First, we applied RepeatScout to predict repetitive consensuses based on a *de novo* genome assembly generated by Velvet. Second, we used wgs-assembler to assemble the same datasets and thereby generate surrogates representing those contigs determined to be repetitive. The respective genome assembly statistics can be found in Supplementary Table S1.

The repeat consensuses were annotated with TEclass (38) (version 2.1) using the default training set that contains oligomer frequencies of all RepBase (release 15.07) repeats. For the purposes of subsequent analyses, a sequence was considered a repeat if it aligned more than once to the genome with at least 80% identity.

### Mapping and repeat masking

All mappings were performed with BLAT (39) (version .34) with default options including ‘−extendThroughN’ to map over stretches of N's and ‘−minIdentity = 50’ to retain lower identity hits. The resulting psl files were further filtered for minimum identity where mentioned in the text. Repeat masking was performed with RepeatMasker (version 4.0.0) with the default parameters and either *D. melanogaster* repeats from RepBase (DmRepBase, release 20120418) or the specified repeat library. For analysis of Alu repeats in the human genome, we extracted 51 Alu consensus sequences from RepBase (release 18.07) categorized as ‘Homo sapiens and Ancestral’, and determined completeness by masking extracted Alu sequences using the RepARK repeat library.

### Retrieving known segmental duplications and comparison to the *de novo* repeat consensuses

We downloaded the positions of SD identified in release 5 of the *D. melanogaster* reference sequence (http://humanparalogy.gs.washington.edu/dm3/dm3wgac.html). SDs were retrieved from the reference genome and masked with DmRepBase such that 3.09 Mb SD regions without RepBase repeats remain. Each repeat library was also masked separately with DmRepBase. The remaining SD sequences were subsequently masked with each masked repeat library to calculate the fraction of SDs each library can identify.

## RESULTS

**Figure 1.**
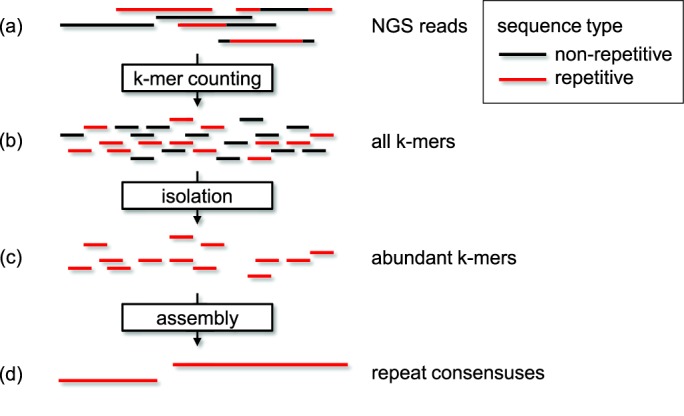
Workflow of the repeat library creation pipeline RepARK. WGS sequencing reads (**a**) contain unique (black) and repetitive (red) fractions of the genome. K-mers of all reads (**b**) were counted and the threshold of frequent k-mers is determined. These abundant k-mers are isolated (**c**) and assembled by a *de novo* genome assembly program (such as Velvet) into repeat consensus sequences (**d**).

A summary of the method to create *de novo* repeat libraries from NGS WGS reads (RepARK) is depicted in Figure 1. To benchmark our approach for the *de novo* creation of repeat libraries, we used the *D. melanogaster* genome due to the availability of a high-quality reference genome (40–41) (version R5.43), an advanced, manually curated repeat library (RepBase Update version 20120418), and NGS WGS reads. For this study, we analyzed both simulated (‘simulated’) and experimentally derived (‘real’) datasets. With simulated data, we know the genomic sequence from which the data is derived, and can therefore ameliorate mis-assemblies in the reference sequence as a source of error in our analyses as well as sequencing biases of the Illumina technology (e.g. underrepresentation of G+C-rich regions (42)). With real data, we can determine whether the method is valid even in the face of real world confounding elements such as technical biases or contaminations.

RepARK libraries were compared against both established repeat libraries and those generated using state-of-the-art methods (Tables 1 and 2). The *D. melanogaster* repeat library of RepBase (DmRepBase) and the ReAS *de novo* repeat library (ReASLib) from the Drosophila 12 genomes project (19) were downloaded as established repeat libraries. RepeatScout was used to generate repeat libraries based on Velvet *de novo* genome assemblies of both simulated and real datasets, while wgs-assembler surrogates are those which have been identified as repeats during assembly graph resolution. Generation of RepARK libraries using either Velvet (RepARK Velvet) or CLC Assembly Cell (RepARK CLC) was orders of magnitude (14×–465×) faster than when using *de novo* state-of-the-art methods. It is notable that the N50 values (the consensus size above which half the total size of the library is represented) of the repeat libraries generated by either RepARK, RepeatScout or wgs-assembler are one to two orders of magnitude (16×–93×) smaller than either the RepBase or ReASLib repeat libraries, indicating extensive fragmentation of the consensuses. The larger total length of libraries created by wgs-assembler and RepARK (2×–7×) in respect to DmRepBase hints to higher redundancies.

**Table 1.** *D. melanogaster* repeat library metrics from simulated NGS reads

	RepeatScout	wgs-assembler	RepARK CLC	RepARK Velvet
Identification method	Velvet + RepeatScout	wgs-assembler surrogates	CLC	Velvet
Number of consensuses	1239	18 203	67 968	14 147
Total length (Mb)	0.174	4.3	4.3	1.9
Min./max. length (bp)	51/2565	66/6446	30/6945	57/6943
N50 (bp)	78	147	58	149
N90 (bp)	64	116	36	59
Time to create (h)	8.75	284	0.61	0.61

**Table 2.** *D. melanogaster* repeat library metrics from real data

	DmRepBase	ReASLib	RepeatScout	wgs-assembler	RepARK CLC	RepARK Velvet
Source data	N/A	Sanger reads	Illumina reads	Illumina reads	Illumina reads	Illumina reads
Identification method	Manual curation	Seed based	Velvet + RepeatScout	wgs-assembler surrogates	CLC	Velvet
Number of consensuses	249	391	414	14 296	19 677	4284
Total length (Mb)	0.7	0.96	0.035	2.2	1.6	0.87
Min./max. length (bp)	52/14 477	101/12 876	51/616	64/25 962	30/7589	57/7587
N50 (bp)	5402	4757	83	158	87	290
N90 (bp)	1750	1247	56	76	38	89
Time to create (h)	N/A	N/A	5.75	101	0.28	0.28

N/A: not applicable

**Figure 2.**
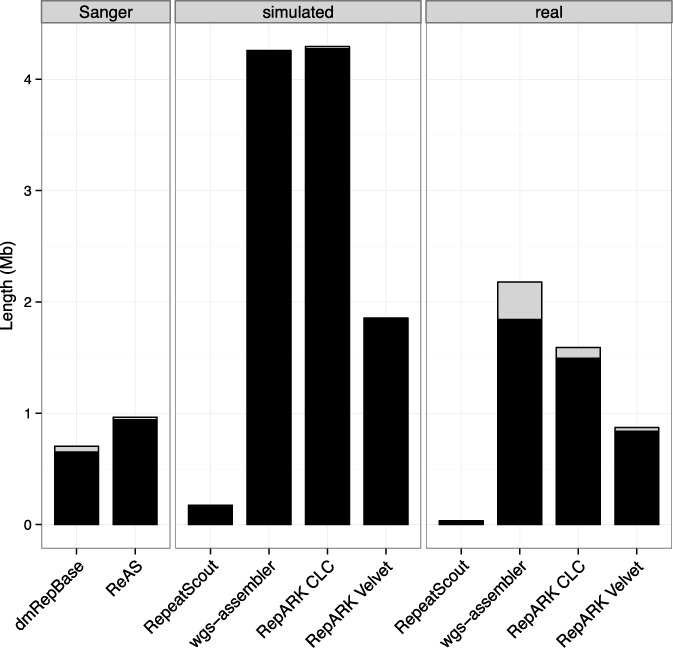
Cumulative length of repetitive and non-repetitive consensuses within each library. Black: repetitive consensuses (i.e. align more than once to the reference); gray: non-repetitive consensuses (i.e. singly mapping or not at all); Sanger: libraries based on Sanger sequencing data; simulated: libraries derived from simulated NGS reads; real: libraries derived from Illumina reads.

To evaluate specificity, each repeat library was mapped onto the *D. melanogaster* genome using BLAT and filtered for minimum identity of 80%. Consensuses encompassing the bulk of each repeat library length (84–99%) mapped multiple times to the reference sequence (henceforth called ‘repetitive consensuses’) (Figure 2, black), while the remaining sequence aligned only once or not at all (Figure 2, gray). A similar fraction of repetitive consensuses were measured for identity thresholds of 90% and 95% for all libraries (Supplementary Table S2). The largest fraction of non-repetitive consensuses was observed in the wgs-assembler library created from real data. Although being composed nearly entirely of repetitive consensuses, the overall length of the RepeatScout library was considerably shorter than the other libraries (Tables 1 and 2, Figure 2). Repeat masking the two assemblies used by RepeatScout revealed that only 6.5% (simulated) and 4.7% (real) of each assembly could be identified as repeats. The vast majority (>99%) of consensuses from RepARK libraries had an average nucleotide coverage >10× (Supplementary Figure S2), and most repetitive consensuses align fewer than 100 times to the reference (Supplementary Figure S3).

**Figure 3.**
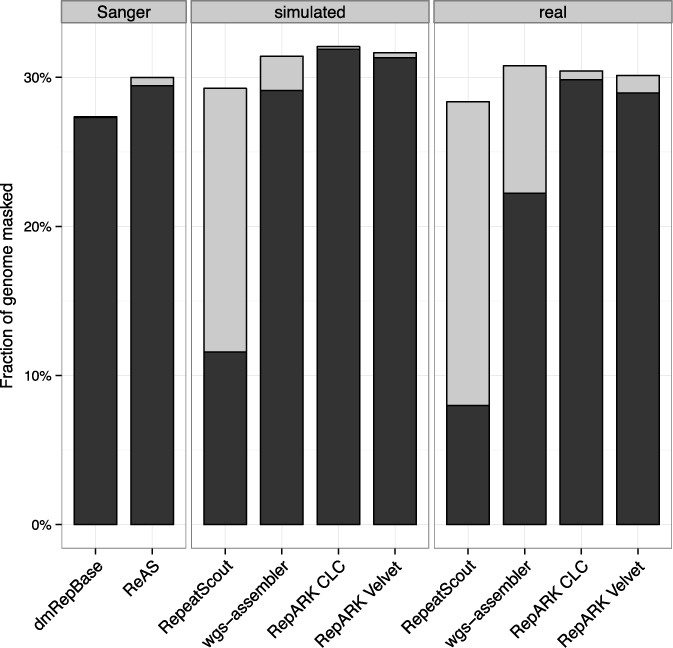
Repeat fractions identified in the *D. melanogaster* reference sequence. Black: fraction of the reference masked by RepeatMasker using the respective repeat library; gray: fraction of the reference that was subsequently masked by RepeatMasker using RepBase; Sanger: libraries based on Sanger sequencing data; simulated: libraries derived from simulated NGS reads; real: libraries derived from Illumina reads.

To evaluate the potential of each library for masking genomic repeats, the *D. melanogaster* reference was masked using RepeatMasker with the corresponding library (Figure 3, black). More of the reference sequence was identified as repetitive when using either the RepARK libraries or ReASLib than when using RepBase. Of state-of-the-art methods, wgs-assembler-based repeat libraries provided comparable results only using simulated reads, while the two RepeatScout derived libraries could mask only a small fraction of the reference. Moreover, when the masked reference is subsequently masked with DmRepBase, only a small fraction of the unmasked genome sequence was identified as repetitive for RepARK libraries (0.18–1.18%) and ReASLib (0.56%) (Figure 3, gray), while wgs-assembler (2.3–8.5%) and RepeatScout (17–20%) derived libraries left much of the repeat fraction of the genome unmasked.

**Figure 4.**
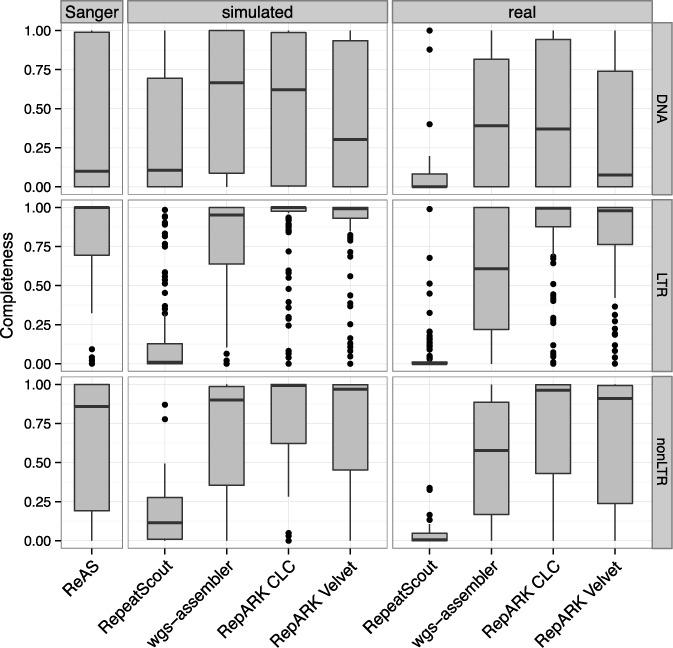
Boxplot of DmRepBase repeat class completeness in the *de novo* repeat libraries. DNA: 33 DNA transposons; LTR: 138 LTR retrotransposons; non-LTR: 41 non-LTR retrotransposons; Sanger: libraries based on Sanger sequencing data; simulated: libraries derived from simulated NGS reads; real: libraries derived from Illumina reads; box: first and third quartiles; horizontal line: median; whiskers: most extreme value within 1.5× of inter-quartile range; dots: outliers. A full table of repeat family representation in the RepARK libraries can be found in Supplementary Table S3.

DmRepBase contains 249 annotated repeat consensuses. Completeness of each of these consensuses in the other repeat libraries was determined by masking them using RepeatMasker and DmRepBase (Figure 4, Supplementary Table S3) and evaluating what fraction of each DmRepBase consensus was used for masking. In general, LTR and non-LTR retrotransposons showed a higher median completeness than DNA transposons. However, RepARK libraries consistently showed as good or superior completeness compared to the other libraries investigated.

**Figure 5.**
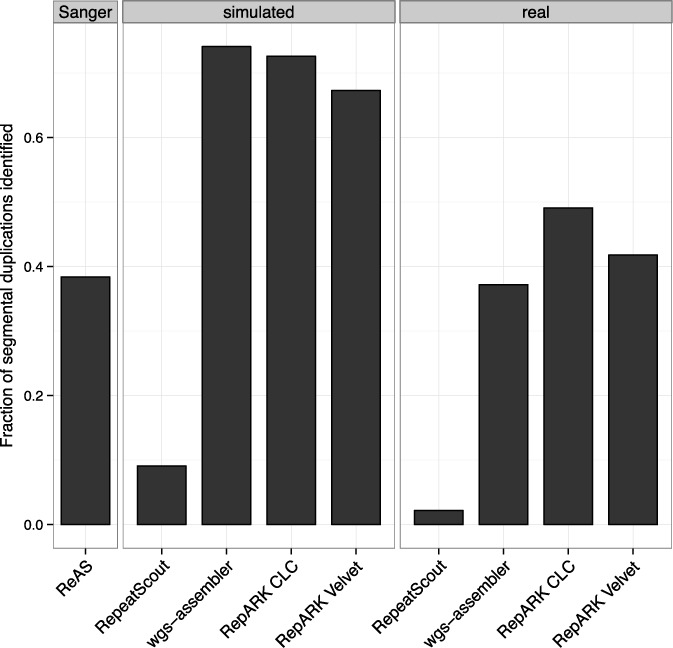
Fractions of known *D. melanogaster* segmental duplications identified by the *de novo* repeat libraries. Sanger: libraries based on Sanger sequencing data; simulated: libraries derived from simulated NGS reads; real: libraries derived from Illumina reads.

Next, we explored potentially novel repeats in each of the *de novo* libraries by mapping the consensuses not recognized as RepBase repeats by RepeatMasker to the *D. melanogaster* reference. Using this approach, we found consensuses that map with high identity proximal to one another on the same chromosome (Supplementary Figure S4) and/or to the corresponding heterochromatin entry (Supplementary Figure S5), patterns characteristic of SDs (10). We therefore retrieved a list of known *D. melanogaster* SDs and determined the fraction identified by those *de novo* library consensuses that were not recognized as DmRepBase repeats. The largest fraction of the SDs could be identified by the RepARK libraries compared to the other *de novo* repeat libraries studied (Figure 5), with the exception of wgs-assembler surrogates using simulated data.

**Figure 6.**
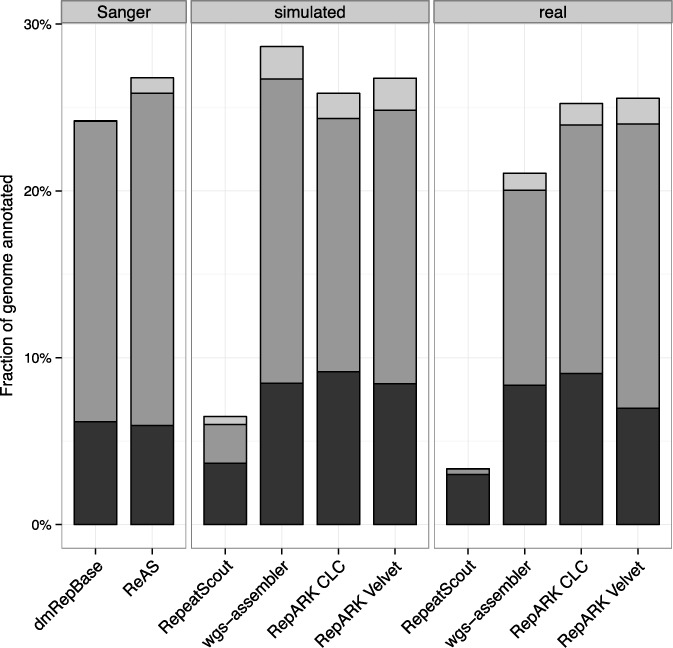
Fractions of the *D. melanogaster* genome reference classified according to annotated repeat libraries. Black: DNA transposon sequence; dark gray: retrotransposon sequence; light gray: unclear; Sanger: libraries based on Sanger sequencing data; simulated: libraries derived from simulated NGS reads; real: libraries derived from Illumina reads.

TEclass, commonly used to annotate repeat libraries, requires consensuses ≥50 bp for classification. In each library analyzed in this study, more than 90% of such consensuses were successfully classified by TEclass. A greater proportion of consensuses in the RepARK libraries were annotated as DNA transposons and fewer as retrotransposons as compared to ReASLib or DmRepBase (Supplementary Table S4), and more of the reference sequence was annotated as DNA transposons at the expenses of retrotransposons using the RepARK libraries (Supplementary Table S5). This bias could be due to the extensive fragmentation of the RepARK libraries to which the TEclass algorithm may not be adopted. Consequently, we restricted the TEclass annotation to consensuses >100 bp, which considerably reduced the bias toward DNA transposons in the repeat annotation of the genome using these RepARK libraries (Figure 6).

Finally, we wanted to determine whether the findings of RepARK as applied to the *D. melanogaster* datasets could be extended to larger, more complex genomes. To this end, we downloaded Illumina read libraries used in the *de novo* assembly of a human genome and generated a RepARK repeat library using the same parameters described previously (Table 3). In this case, we utilized Velvet due to its frequent use in academic environments. The RepARK library (7.9 Mb) was again substantially longer than the human RepBase repeat library (HsRepBase, 1.6 Mb), and a similar fraction of the cumulative length of the human RepARK library was found to be composed of repetitive consensuses (93%) as in that for *D. melanogaster* (Figure 2). Additionally, 37 of 51 of the highly abundant and mobile Alu families were at least 50% represented within the RepARK library (Supplementary Table S6).

**Table 3.** Human repeat library metrics and mapping results against the human reference sequence

	HsRepBase	RepARK Velvet
Number of consensuses	1439	62 425
Total length (kb)	1566	7882
Min./max. length (bp)	63/9044	57/42 518
N50 (bp)	2822	143
N90 (bp)	471	57
Time to create (hrs)	N/A	22
Number of consensuses with multiple hits	1167 (81%^a^)	57 239 (92%^a^)
Total length of consensuses with multiple hits (kb)	1471 (94%^b^)	7318 (93%^b^)

^a^Ratio to the total number of consensuses of the library.

^b^Ratio to the total length of the library.

**Figure 7.**
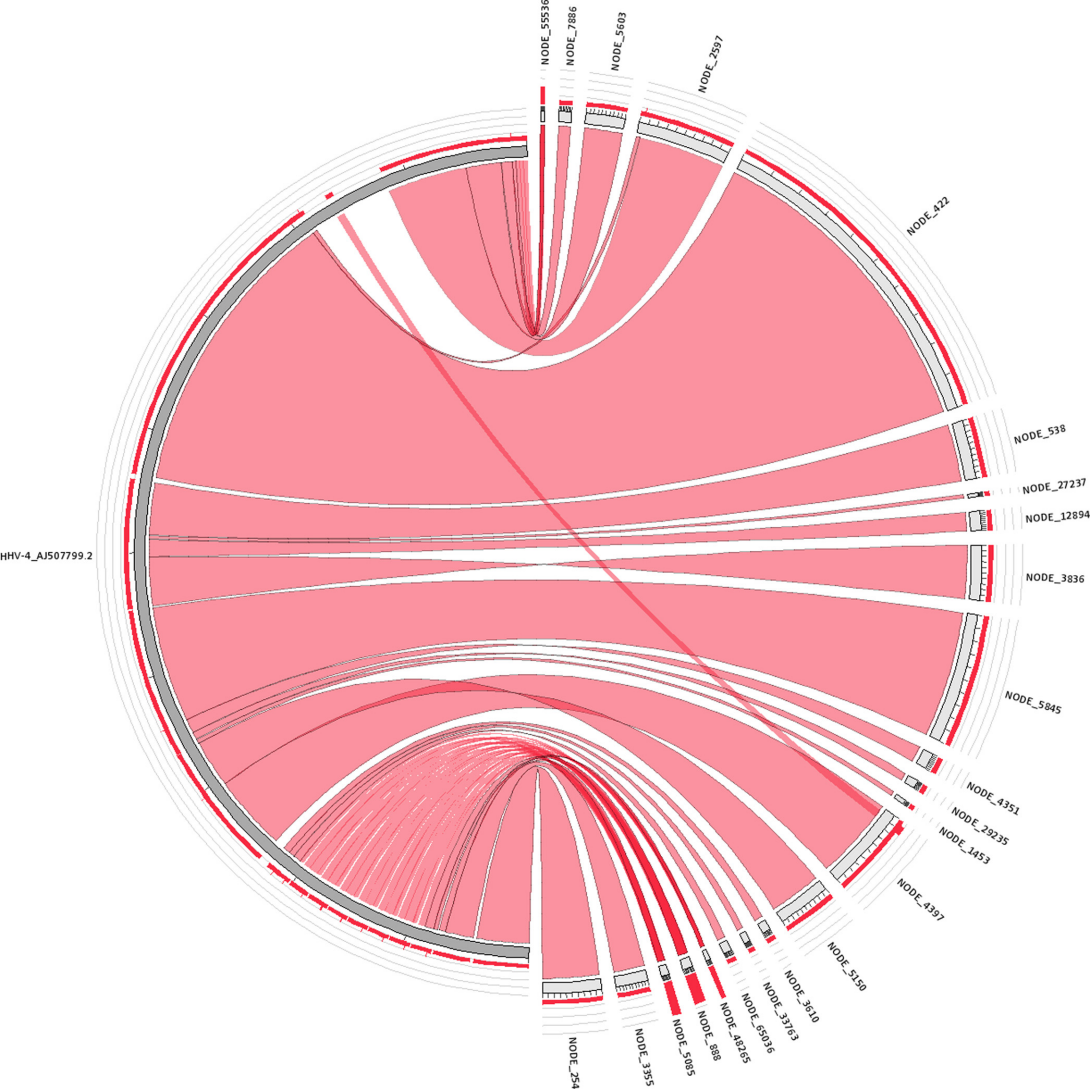
High confidence alignments of human RepARK consensuses (right half) to the Epstein-Barr virus genome (left half, HHV-4). Each ribbon represents a consensus alignment with >90% mapping and *p* < 10^−60^, encompassing 90.5% of the Epstein-Barr virus genome. Lower confidence consensuses align to the remaining 9.5% with more relaxed criteria. Three consensuses map multiple times to the virus genome sequence (NODE_48265, NODE_888, NODE_5085; dark red). Created with Circoletto (http://bat.ina.certh.gr/tools/circoletto/).

Surprisingly, RepARK also generated a number of very long consensuses from the human NGS data, the longest being 42518 bp (almost twice as long as the longest known LTR retrotransposon *ogre* with 25 kb (43)). Aligning this consensus with BLAST against ‘Nucleotide collection (nt/nr)’ (http://blast.ncbi.nlm.nih.gov) identified a highly significant match to the Epstein-Barr virus (EBV alias Human herpes virus 4, HHV-4) which was used to establish the human cell line sequenced (Coriell Institute, GM12878). After further investigation, 23 repeat consensuses were identified with >90% of their bases mapping and *p* < 10^−60^ to the EBV genome. The majority (90.5%) of the 171 kb virus genome is covered by one of the consensuses using these parameters (Figure 7), and the remaining 9.5% is covered by consensuses using more relaxed criteria.

## DISCUSSION

Generation of repeat libraries is an important step for accurate analyses of genomes, but has historically relied heavily upon manual curation (44). With the availability of genome assemblies and NGS, new prediction models came into practice (reviewed in (45)). These approaches are dependent on the quality of the genome sequence analyzed, and assemblers using short reads from NGS technologies are notoriously poor at resolving repetitive genomic segments due to the length and complexity of genomic repeats. As an alternative to a reference-based approach, we describe here RepARK, a novel, NGS-based method for building and annotating a library of repeat consensuses without a reference genome. This method relies on k-mer counting, a routine step in sequence analysis (26). After counting, k-mers predicted to occur more than once genome-wide (‘abundant’) are *de novo* assembled with a de Bruijn graph assembler and a comprehensive repeat library is generated.

For the proof-of-principle, we selected the *D. melanogaster* genome for its moderate size and repeat content and for the high-quality reference sequence available (40–41). We validated the method on both simulated and experimentally derived data using both commercial (CLC) and open source (Velvet) de Bruijn graph assemblers. The overall lengths of the RepARK repeat libraries are longer than that found in RepBase (0.87–4.3 Mb versus 0.7 Mb), and >90% of consensuses in all RepARK libraries are repetitive. Moreover, only a small fraction of the reference masked with a RepARK library can be subsequently identified by RepBase as a repeat (0.18–1.18%), indicating that the bulk of RepBase repeats in the genome can be identified using the RepARK method. Although we required a sequence identity of >80% for mapping of the consensuses to the reference (the standard threshold for the identification of a repeat motif), the number of RepARK library repetitive consensuses did not change even with a threshold of >90% or >95% (Supplementary Table S2), most likely due to the sequence fragmentation in the de Bruijn graph. The high ratio of consensus length and greater overall consensus length that maps more than once to the reference in the RepARK libraries indicates that the presented method may generate genome-specific repeat libraries with comparable or even higher sensitivity and specificity than the RepBase approach that is focused on the identification and reconstruction of genome-wide dispersed transposons and does not tackle, e.g., SDs.

Although wgs-assembler using simulated data produced a comprehensive repeat library in almost all metrics examined in this study, these positive results were not reflected when using a real dataset. In particular, while the repeat library derived from real data contained repetitive consensuses with a longer total length compared to the other libraries, it was substantially less effective in masking the reference genome (22% versus 27–32% for the other non-RepeatScout libraries) This discrepancy between length of repetitive consensuses and length of the reference masked could be due to consensus redundancy. It is also important to note that the RepeatScout-based method, arguably the most popular state-of-the-art method for *de novo* generation of repeat libraries, was the least effective at generating comprehensive repeat libraries of all the methods examined. The fact that a low completeness of repeats could be identified in the Velvet-based genome assemblies only underscores the reliance of RepeatScout on a high-quality draft reference assembly that is frequently difficult to obtain using only NGS libraries. In the course of preparing this publication, a novel *D. melanogaster* assembly was reported that has been derived from >90× coverage by reads obtained using the PacBio technology with an average length of 10 kb (http://blog.pacificbiosciences.com/2014/01/data-release-preliminary-de-novo.html). In this assembly, PacBio reads resolve unique repetitive transposable elements up to ∼10 kb in size, indicating that long reads may also provide new opportunities for *de novo* repeat prediction. Finally, the RepARK method is orders of magnitude faster than the state-of-the-art methods due to assembly graph simplification, making RepARK a useful tool for prototyping reference repeat libraries as well as generating repeat libraries for individual samples. While the ReAS library was comparable in almost every metric evaluated to RepARK libraries and uses a similar method to generate repeat libraries, it requires labor- and cost-intensive Sanger-type long sequences and is unable to deal with short NGS reads. In point of fact, we were not able to evaluate ReAS using either our simulated or real read data due to the limitations of the program.

More consensuses were found in RepARK libraries from the simulated dataset than from the real data (Table 1). Such a discrepancy could result from assembly errors in the reference sequence leading to an artificial overrepresentation of certain motifs. This explanation is supported by noting that the U and Uextra chromosomes, included as templates for read simulation, are hotspots for assembly errors (46). Alternatively, real sequencing data are subjected to various technological biases leading to the underrepresentation of particular motifs (e.g. GC-rich or heterochromatin sequence, both regions of high repeat content (42)). Finally, it is possible that this discrepancy is due to actual genomic differences between the reference and the DNA sample sequenced such as copy number variation or SDs.

Although we observe RepBase consensuses with a completeness of <50%, only ∼1% of the RepARK library-masked *D. melanogaster* reference genome could be subsequently masked using RepBase (Figure 3, gray). It is particularly telling that one-third of such consensuses belong to the RepBase group ‘remaining’, which contains consensus annotation such as ‘ARTEFACT’. Such consensuses are derived from cloning artifacts and would therefore not be detected using cloning-free NGS methods. Moreover, the DmRepBase library contains ancestral repeat consensuses that may not be repetitive or represented at all in the reference genome and therefore could not be detected as repeats by RepARK. Alternatively, some of the very short RepARK consensuses may not be usable by RepeatMasker when masking the DmRepBase library resulting in underestimation of completeness. Finally, highly divergent repeat motifs may cause excessive fragmentation of the assembly graph, the consensuses of which may be lost by our size cutoff of 50bp. This seems a likely scenario given the high fraction of short consensuses within the RepARK libraries and could be at least partially rectified by using a *de novo* assembler that uses more relaxed criteria for calling consensus sequences.

More of the genome is masked by RepeatMasker using the RepARK libraries than with DmRepBase (1.6–4.5% additional sequence). Part of this additional masked sequence can be explained by the observation that a portion of the RepARK consensuses represents SDs, which can be specific for individual genomes. Such a finding is compatible with the fact that RepBase libraries contain only simple and genome-wide dispersed repeats. To date, SDs are detected using traditional whole-genome alignment methods based on criteria that exclude shorter, more divergent sequences (<90% identity, <1 kb). This limitation could explain some of the putative novel SD events identified using the RepARK libraries, such as that observed for chromosome X (Supplementary Figure S4). Additionally, the use of whole-genome alignments to detect SDs runs the risk of false positives/negatives due to assembly errors in the reference sequence. Together with the high ratio of fully mappable consensuses, these data further underpin the conclusion that the consensuses produced by RepARK are both highly specific and sensitive for detection of repetitive elements of a given genome.

The bias toward DNA transposon annotation by TEclass for the NGS *de novo* libraries represents a limitation for accurately annotating repeat classes in a genome. This behavior is most likely due to the highly fragmented nature of such libraries, which may present a challenge for some of the annotation models implemented in TEclass. Revising these models may produce more accurate annotation of highly fragmented repeat libraries such as those investigated in this study. Alternatively, creation of longer repeat consensuses (such as that found in RepARK library generated by Velvet) or the restriction of the TEclass library annotation to longer consensuses (>100 bp) can also improve repeat annotation. Regardless to further improvements, precise examination of repeat evolution in newly assembled genomes will require closer, manual examination. Nevertheless, the consensuses of NGS *de novo* libraries can be used to identify and isolate repetitive genomic elements with high accuracy and to provide a first pass annotation.

The high rate of true positives and long overall length seen for *D. melanogaster* RepARK libraries was also found in the human RepARK library, indicating that this method is readily extensible to larger and more complex genomes. Alu repeat elements are high-frequency retrotransposons that are still mobile within the human genome (47), and a majority of Alu families were represented by more than 50% in the RepARK repeat library. Unexpectedly, the entire EBV genome was found within the RepARK library, a finding that can be readily explained by noting that EBV was used to establish the cell line from which the human DNA was isolated and sequenced. As EBV generally does not integrate into the host chromosomes, it exists as a circular episome within the nucleus (see review (48)). This finding suggests that RepARK may also represent a novel method to quickly identify contaminants within a DNA dataset and may find future application not only as a repeat library generator, but also as a diagnostic tool.

Taken together, our k-mer-based method can use sequences as short as 31 bp, is independent of an assembled genome sequence, can utilize any de Bruijn assembler, generates consensuses for which the vast majority are repetitive and can be annotated by TEclass. It can be applied to genomes at least as large and complex as the human genome. Construction of these libraries is orders of magnitude faster and represents a new approach to identify SDs, multi-copy contaminations or pathogens directly from NGS datasets. Finally, we showed that RepARK repeat libraries are as good as or better than that of the state-of-the-art methods examined.

## SUPPLEMENTARY DATA

Supplementary Data are available at NAR Online.

## DATA ACCESS

The generated repeat libraries can be downloaded from ftp://genome.fli-leibniz.de/pub/repeat-assemblies/ and the RepARK script via https://github.com/PhKoch/RepARK.

## Supplementary Material

SUPPLEMENTARY DATA
