# Supplementary material for: RepARK—de novo creation of repeat libraries from whole-genome NGS reads
Source: Nucleic Acids Res. 2014 Mar 14;42(9):e80. doi: 10.1093/nar/gku210 (PMC4027187; doi:10.1093/nar/gku210)
Supplement: SUPPLEMENTARY DATA [file supp_42_9_e80__index.html]

RepARK—de novo creation of repeat libraries from whole-genome NGS reads — SUPPLEMENTARY DATA 

# RepARK—*de novo* creation of repeat libraries from whole-genome NGS reads

## SUPPLEMENTARY DATA

**Files in this Data Supplement:**

- SUPPLEMENTARY DATA
