# Supplementary material for: RepARK—de novo creation of repeat libraries from whole-genome NGS reads
Source: Nucleic Acids Res. 2014 Mar 14;42(9):e80. doi: 10.1093/nar/gku210 (PMC4027187; doi:10.1093/nar/gku210)
Supplement: SUPPLEMENTARY DATA [file supp_gku210_nar-01653-met-k-2013-File010.pdf]

RepARK - *de novo* creation of repeat libraries from whole-genome NGS reads

Philipp Koch, Matthias Platzer and Bryan R. Downie

Supplementary Figures

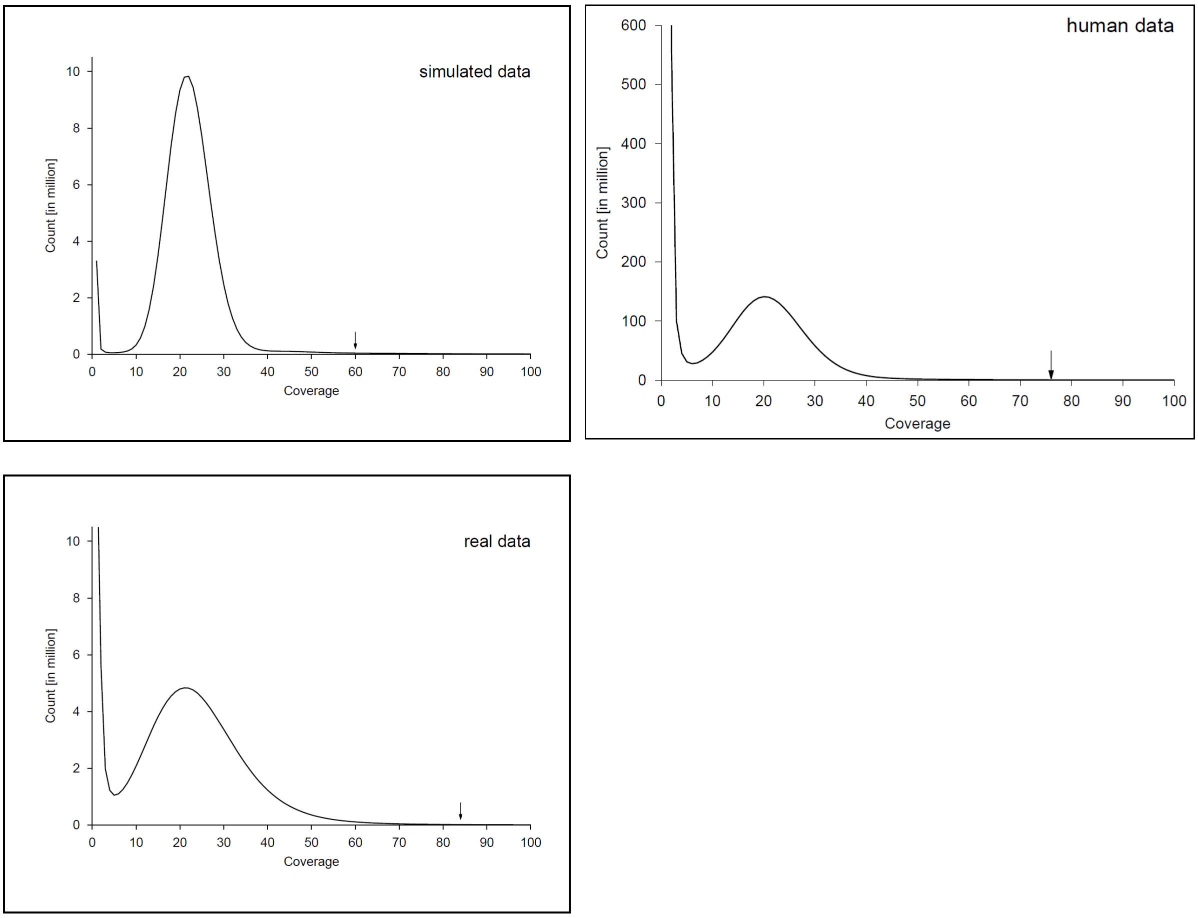

Supplementary Figure 1. 31-mer coverage histograms for simulated (top left) and real (bottom left) *D. melanogaster* and real human (top right) Illumina reads. Arrows indicate the thresholds defining 31-mers as abundant (simulated: >60, real: >84, human: 76).

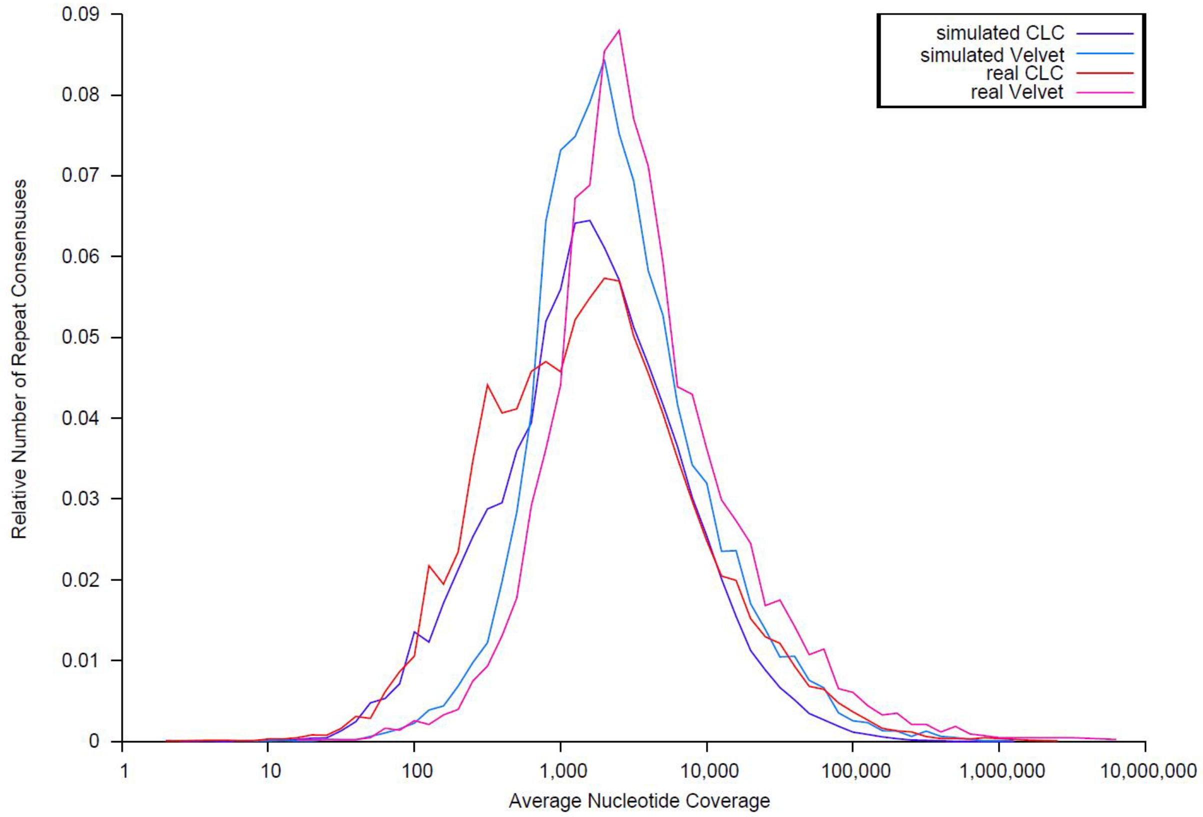

Supplementary Figure 2 Histogram of nucleotide coverage of the RepARK libraries. Average nucleotide coverage (avg_c) for consensuses was calculated by mapping k-mers of length 31 ($length_{k\text{-}mer}$) with an identity of 100% and mapping proportion of 100% onto the consensuses and applying the following formula:

$$avg_c = \frac{\sum occ_{k-mer} \times length_{k-mer}}{length_{consensus}}$$

where $occ_{k\text{-}mer}$ represents the number of occurrences of a mappable k-mer in the entire data set.

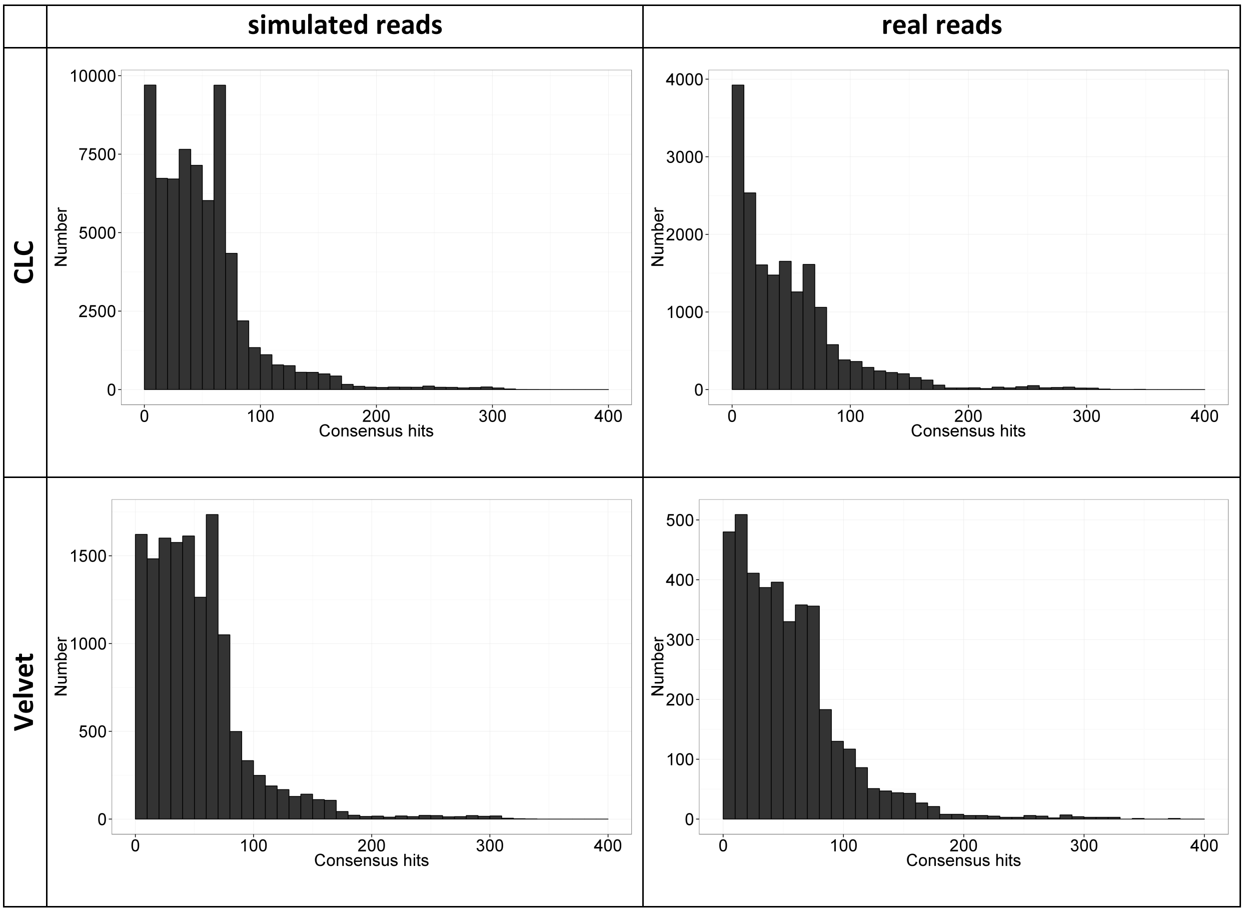

Supplementary Figure 3: Histograms of repetitive consensus copy number for RepARK libraries to the *D. melanogaster* reference genome. Repeat consensuses were aligned with BLAT and 80% minimum identity as described in Methods. Note that the number of consensuses on the y-axes differ for each library.

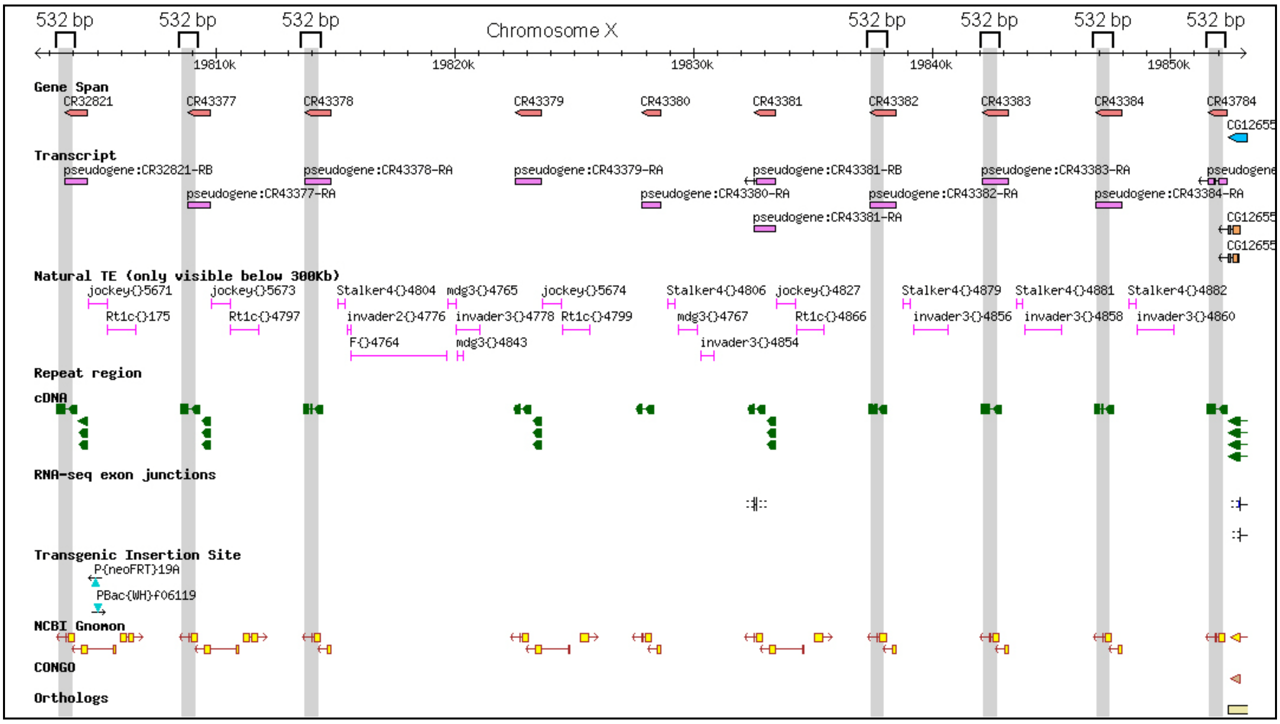

Supplementary Figure 4. Example of short length putative segmental duplication event. A 532 bp consensus from the real RepARK CLC library shows a pattern of SD (gray shaded) on chromosome X (100% identity). The snapshot was taken from flybase.org; chromosomal location and consensus lengths were added.

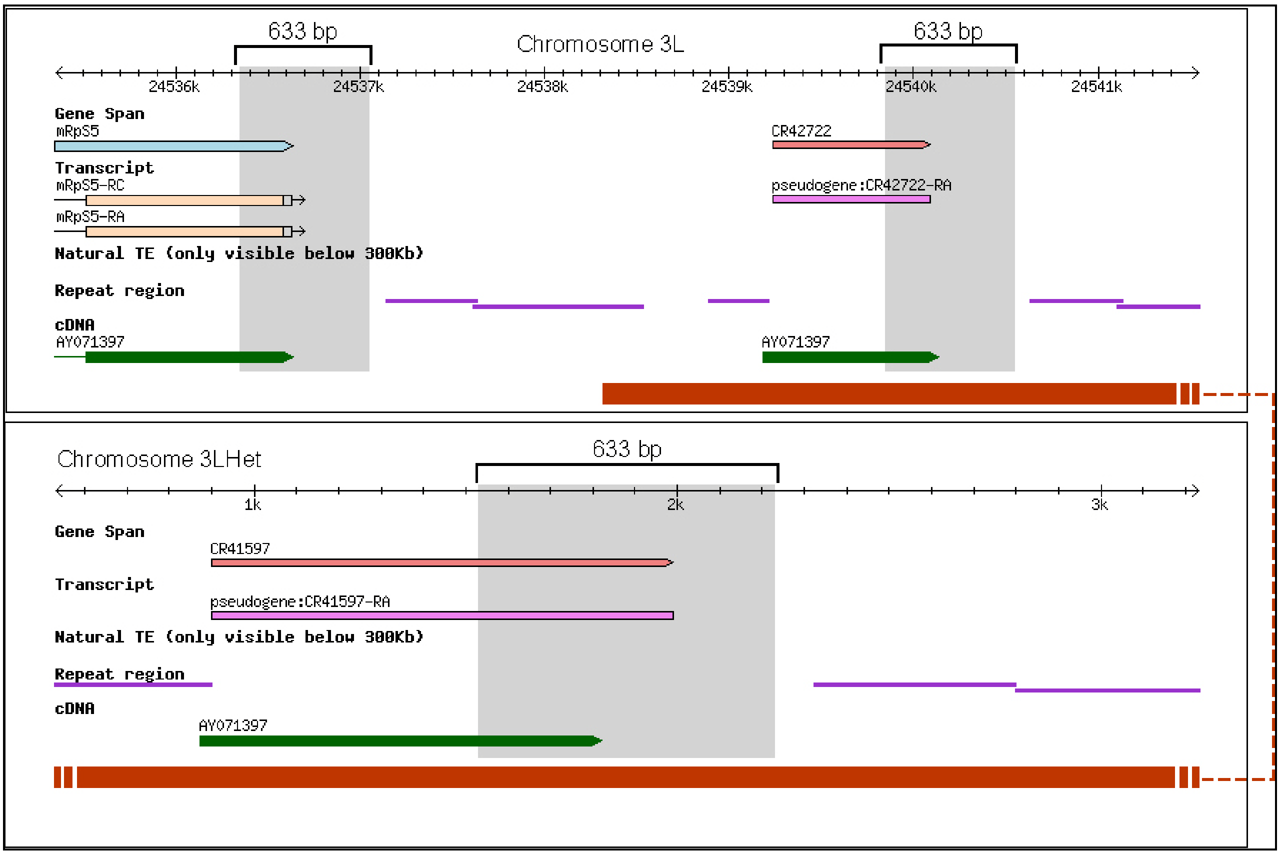

Supplementary Figure 5. Example of segmental duplications involving heterochromatin identified by the NGS *de novo* libraries.

A consensus from the simulated RepARK CLC library shows a pattern of SD (gray shaded) within chromosome 3L. This particular consensus also maps to the 3L heterochromatin. Red bar (4.4 kb) represents a pair of a SD detected by Eichler et al. The snapshot was taken from flybase.org; chromosomal locations, consensus lengths and SD annotation were added.

Supplementary Tables

Supplementary Table 1. Statistics of whole genome *de novo* assemblies of *D. melanogaster*.

	simulated reads		real reads	
Asssembler	Velvet	wgs-assembler	Velvet	wgs-assembler
Number of sequences	66,720	1,348	47,680	4,045
Total length	126.7 Mb	121.8 Mb	117.7 Mb	116.7 Mb
Min/max length	57 bp / 1.3 Mb	103 bp / 1.4 Mb	57 bp / 0.3 Mb	166 bp / 434 kb
Average	1,899 bp	90 kb	2,468 bp	29 kb
N50	276 kb	328 kb	9 kb	73 kb
N90	19 kb	54 kb	4 kb	13 kb

Supplementary Table 2. BLAT mapping of repeat libraries with 80%, 90% and 95% mapping identity to the *D. melanogaster* genome.

	Library	Number of hits			Consensuses with >1 hit			Consensuses with >1 hit including at least one hit to U/Uextra chromosome		
		80%	90%	95%	80%	90%	95%	80%	90%	95%
Sanger	DmRepBase	20,627	17,241	12,302	213 (86%)[a]	212 (85%)	209 (84%)	209 (84%)	207 (83%)	197 (79%)
Sanger	ReASLib	38,734	34,539	23,639	342 (87%)	342 (87%)	341 (87%)	313 (80 %)	313 (80%)	307 (79%)
simulated reads	Repeat Scout	50,609	47,897	29,857	1,236 (100%)	1,234 (100%)	1,207 (97%)	972 (78%)	965 (78%)	900 (73%)
simulated reads	wgs-assembler	1.5 M	1.4 M	1.0 M	18,165 (100%)	18,164 (100%)	18,133 (100%)	17,234 (95%)	17,188 (94%)	16,691 (92%)
simulated reads	RepARK CLC	3.4 M	3.3 M	2.9 M	67,419 (99%)	67,418 (99%)	67,407 (99%)	55,310 (81%)	55,120 (81%)	53,160 (78%)
simulated reads	RepARK Velvet	0.7 M	0.7 M	0.5 M	14,141 (100%)	14,141 (100%)	14,141 (100%)	12,869 (91%)	12,796 (90%)	12,077 (85%)
real reads	Repeat Scout	19,147	18,330	11,447	411 (99%)	409 (99%)	391 (94%)	325 (79%)	321 (78%)	272 (66%)
real reads	wgs-assembler	1.1 M	1.1 M	0.9 M	10,627 (74%)	10,625 (74%)	10,571 (74%)	10,108 (71%)	10,086 (71%)	9,798 (69%)
real reads	RepARK CLC	0.9 M	0.8 M	0.7 M	18,093 (92%)	18,088 (92%)	18,047 (92%)	16,050 (82%)	16,014 (81%)	15,698 (80%)
real reads	RepARK Velvet	0.2 M	0.2 M	0.2 M	4,050 (95%)	4,048 (94%)	4,035 (94%)	3,791 (88%)	3,770 (88%)	3,675 (86%)

[a] Percentages refer to the total number of consensuses within a library. M = million.

Supplementary Table 3. List of repeat consensuses from RepBase with their full names, classes and completeness values within the RepARK libraries.

RepBase consensus name	completeness[a] of RepBase repeats in the library: CLC simulated	Velvet simulated	CLC real	Velvet real	full class name	simple class name
DNAREP1_DM	1	1	1	1	DNA/TcMar-Tc1	DNA
POGON1	1	1	1	1	DNA/TcMar-Tc1	DNA
BARI_DM	1	0.9977	0.9977	0.9977	RC/Helitron	DNA
TRANSIB2	1	0.9975	0.976	0.9546	DNA/TcMar-Tc1	DNA
POGO	0.9993	0.9861	0.974	0.943	DNA/P	DNA
M4DM	0.9977	0.9756	0.9582	0.9233	RC/Helitron	DNA
S_DM	0.9975	0.9658	0.9466	0.8618	DNA/PiggyBac	DNA
PROTOP_B	0.9974	0.9464	0.9462	0.752	DNA/CMC-Transib	DNA
PROTOP_A	0.9866	0.9349	0.943	0.7394	DNA/TcMar-Mariner	DNA
PROTOP	0.943	0.8773	0.909	0.6388	DNA/TcMar-Tc1	DNA
TC1_DM	0.9424	0.8133	0.8299	0.6008	DNA/TcMar-Tc1	DNA
FB4_DM	0.9298	0.6538	0.8114	0.4324	DNA/TcMar-Tc1	DNA
Transib5	0.8056	0.6525	0.7372	0.3296	DNA/MULE-NOF	DNA
TC1-2_DM	0.794	0.6485	0.572	0.3047	DNA/P	DNA
S2_DM	0.7911	0.4912	0.4658	0.1314	DNA/P	DNA
TRANSIB3	0.6651	0.3284	0.3902	0.0874	DNA/TcMar-Tc1	DNA
Mariner2_DM	0.6214	0.3029	0.3701	0.0757	DNA/P	DNA
NOF_FB	0.4437	0.2685	0.1167	0.0184	DNA/TcMar-Pogo	DNA
BARI1	0.4202	0.2342	0.0697	0	DNA/TcMar-Pogo	DNA
Galileo_DM	0.2648	0.2259	0.0324	0	DNA/P	DNA
Helitron1_DM	0.2424	0.0526	0.0303	0	DNA/P	DNA
LOOPER1_DM	0.1937	0.0505	0.0257	0	DNA/P	DNA
MARINA	0.0828	0.0361	0.0169	0	DNA/TcMar-Tc1	DNA
Mariner_DMA	0.0569	0.0336	0	0	DNA/TcMar-Tc1	DNA
MINOS	0.0048	0	0	0	DNA/TcMar-Tc1	DNA
P_DB	0	0	0	0	DNA/TcMar-Tc1	DNA
P_DG	0	0	0	0	DNA/CMC-Transib	DNA
PARIS	0	0	0	0	DNA/CMC-Transib	DNA
PLACW_DM	0	0	0	0	DNA/CMC-Transib	DNA
TRANSIB1	0	0	0	0	DNA/CMC-Transib	DNA
TRANSIB4	0	0	0	0	DNA/CMC-Transib	DNA
Transib-N1_DM	0	0	0	0	DNA/CMC-Transib	DNA
UHU	0	0	0	0	DNA/TcMar-Tc1	DNA
DOC6_DM	1	1	1	1	LINE/Jockey	LINE
FW_DM	1	1	1	1	LINE/Jockey	LINE
R2_DM	1	1	1	0.9997	LINE/R2	LINE
DOC	1	1	1	0.9996	LINE/Jockey	LINE
DMRT1B	1	1	1	0.9983	LINE/R1	LINE
BS	1	0.9995	1	0.9982	LINE/Jockey	LINE
BS2	1	0.9994	0.9998	0.9979	LINE/Jockey	LINE

LINEJ1_DM	1	0.9993	0.9994	0.9964	LINE/Jockey	LINE
R1_DM	1	0.9992	0.9984	0.9961	LINE/R1	LINE
G6_DM	1	0.9989	0.9983	0.9946	LINE/Jockey	LINE
IVK_DM	0.9996	0.9985	0.9979	0.9937	LINE/I	LINE
I_DM	0.9996	0.9982	0.9964	0.99	LINE/I	LINE
DMCR1A	0.9994	0.9981	0.9946	0.9828	LINE/CR1	LINE
G_DM	0.9994	0.9968	0.9945	0.9816	LINE/Jockey	LINE
DOC2_DM	0.9992	0.9955	0.9939	0.9614	LINE/Jockey	LINE
DMRT1A	0.999	0.9941	0.9931	0.9576	LINE/R1	LINE
HELENA_RT	0.9986	0.9925	0.9836	0.937	LINE/Jockey	LINE
BS3_DM	0.9985	0.9875	0.9816	0.9196	LINE/Jockey	LINE
Baggins1	0.9975	0.9848	0.9812	0.9195	LINE/LOA	LINE
G2_DM	0.9952	0.9766	0.9709	0.911	LINE/Jockey	LINE
DOC3_DM	0.9929	0.9688	0.9618	0.9101	LINE/Jockey	LINE
DOC5_DM	0.9791	0.9629	0.9476	0.836	LINE/Jockey	LINE
TART	0.9773	0.9218	0.8366	0.6282	LINE/Jockey	LINE
HETA	0.9449	0.9024	0.825	0.6274	LINE/telomeric	LINE
TAHRE	0.9274	0.8356	0.7275	0.5966	LINE/Jockey	LINE
G5_DM	0.877	0.7938	0.7028	0.5031	LINE/Jockey	LINE
Jockey2	0.7958	0.6456	0.6913	0.4279	LINE/Jockey	LINE
G5A_DM	0.7583	0.6229	0.6723	0.3985	LINE/Jockey	LINE
G4_DM	0.709	0.5816	0.5501	0.3817	LINE/Jockey	LINE
TART_B1	0.6529	0.4749	0.4826	0.262	LINE/Jockey	LINE
FW2_DM	0.6226	0.452	0.4294	0.2381	LINE/Jockey	LINE
G3_DM	0.5041	0.4398	0.3627	0.2067	LINE/Jockey	LINE
DMRT1C	0.4777	0.4171	0.3483	0.1953	LINE/R1	LINE
DOC4_DM	0.4042	0.2369	0.3096	0.1602	LINE/Jockey	LINE
Bilbo	0.3128	0.2066	0.1443	0	LINE/LOA	LINE
BS4_DM	0.2821	0.1957	0.0656	0	LINE/Jockey	LINE
FW3_DM	0.0491	0	0	0	LINE/Jockey	LINE
G7_DM	0.031	0	0	0	LINE/Jockey	LINE
R1-2_DM	0	0	0	0	LINE/R1	LINE
TRIM	0	0	0	0	LINE/LOA	LINE
Copia_LTR	1	1	1	1	LTR/Copia	LTR/Copia
Copia1-I_DM	1	1	1	1	LTR/Copia	LTR/Copia
Copia1-LTR_DM	1	1	1	1	LTR/Copia	LTR/Copia
Copia2_I-int	1	1	1	1	LTR/Copia	LTR/Copia
Copia2_LTR_DM	1	1	1	1	LTR/Copia	LTR/Copia
DM1731_I-int	0.9949	1	1	1	LTR/Copia	LTR/Copia
DM1731_LTR	0.9909	0.9734	1	1	LTR/Copia	LTR/Copia
FROGGER_LTR	0.9826	0.8623	0.9951	0.9951	LTR/Copia	LTR/Copia
Copia_I-int	0.9746	0.798	0.9826	0.9826	LTR/Copia	LTR/Copia
FROGGER_I-int	0.8474	0.3674	0.7761	0.6649	LTR/Copia	LTR/Copia
ACCORD_I-int	1	1	1	1	LTR/Gypsy	LTR/Gypsy
ACCORD_LTR	1	1	1	1	LTR/Gypsy	LTR/Gypsy
ACCORD2_LTR	1	1	1	1	LTR/Gypsy	LTR/Gypsy

Bica_LTR	1	1	1	1	LTR/Gypsy	LTR/Gypsy
BLASTOPIA_I-int	1	1	1	1	LTR/Gypsy	LTR/Gypsy
BLASTOPIA_LTR	1	1	1	1	LTR/Gypsy	LTR/Gypsy
BLOOD_LTR	1	1	1	1	LTR/Gypsy	LTR/Gypsy
BURDOCK_I-int	1	1	1	1	LTR/Gypsy	LTR/Gypsy
BURDOCK_LTR	1	1	1	1	LTR/Gypsy	LTR/Gypsy
Chimpo_LTR	1	1	1	1	LTR/Gypsy	LTR/Gypsy
Chouto_LTR	1	1	1	1	LTR/Gypsy	LTR/Gypsy
DM297_LTR	1	1	1	1	LTR/Gypsy	LTR/Gypsy
DMLTR5	1	1	1	1	LTR/Gypsy	LTR/Gypsy
Gypsy10_LTR	1	1	1	1	LTR/Gypsy	LTR/Gypsy
Gypsy1-LTR_DM	1	1	1	1	LTR/Gypsy	LTR/Gypsy
Gypsy2-LTR_DM	1	1	1	1	LTR/Gypsy	LTR/Gypsy
Gypsy4_LTR	1	1	1	1	LTR/Gypsy	LTR/Gypsy
Gypsy5_LTR	1	1	1	1	LTR/Gypsy	LTR/Gypsy
Gypsy6A_LTR	1	1	1	1	LTR/Gypsy	LTR/Gypsy
IDEFIX_LTR	1	1	1	1	LTR/Gypsy	LTR/Gypsy
Invader1_I-int	1	1	1	1	LTR/Gypsy	LTR/Gypsy
Invader1_LTR	1	1	1	1	LTR/Gypsy	LTR/Gypsy
Invader2_LTR	1	1	1	1	LTR/Gypsy	LTR/Gypsy
Invader3_I-int	1	1	1	1	LTR/Gypsy	LTR/Gypsy
Invader3_LTR	1	1	1	1	LTR/Gypsy	LTR/Gypsy
Invader4_LTR	1	1	1	1	LTR/Gypsy	LTR/Gypsy
MDG1_LTR	1	1	1	1	LTR/Gypsy	LTR/Gypsy
MDG3_LTR	1	1	1	1	LTR/Gypsy	LTR/Gypsy
MICROPIA_I-int	1	1	1	1	LTR/Gypsy	LTR/Gypsy
MICROPIA_LTR	1	1	1	1	LTR/Gypsy	LTR/Gypsy
ROVER-LTR_DM	1	1	1	1	LTR/Gypsy	LTR/Gypsy
Stalker2_LTR	1	1	1	1	LTR/Gypsy	LTR/Gypsy
STALKER4_LTR	1	1	1	1	LTR/Gypsy	LTR/Gypsy
TABOR_LTR	1	1	1	1	LTR/Gypsy	LTR/Gypsy
TIRANT_LTR	1	1	1	1	LTR/Gypsy	LTR/Gypsy
TLD2	1	1	1	1	LTR/Gypsy	LTR/Gypsy
TRANSPAC_I-int	1	1	1	1	LTR/Gypsy	LTR/Gypsy
TRANSPAC_LTR	1	1	1	1	LTR/Gypsy	LTR/Gypsy
IDEFIX_I-int	1	1	1	0.9998	LTR/Gypsy	LTR/Gypsy
Invader2_I-int	1	1	1	0.9987	LTR/Gypsy	LTR/Gypsy
Gypsy4_I-int	1	0.9998	1	0.9963	LTR/Gypsy	LTR/Gypsy
Gypsy7_LTR	1	0.9997	1	0.996	LTR/Gypsy	LTR/Gypsy
QUASIMODO_I-int	1	0.9996	1	0.9942	LTR/Gypsy	LTR/Gypsy
Bica_I-int	1	0.9994	1	0.9941	LTR/Gypsy	LTR/Gypsy
MDG3_I-int	1	0.999	1	0.9936	LTR/Gypsy	LTR/Gypsy
DM297_I-int	1	0.9989	1	0.9935	LTR/Gypsy	LTR/Gypsy
BLOOD_I-int	1	0.9975	1	0.9933	LTR/Gypsy	LTR/Gypsy
DM412	1	0.9974	0.9991	0.9921	LTR/Gypsy	LTR/Gypsy

DM412B_LTR	1	0.9966	0.999	0.9877	LTR/Gypsy	LTR/Gypsy
Gypsy_I-int	1	0.996	0.9983	0.9866	LTR/Gypsy	LTR/Gypsy
CIRCE	1	0.9955	0.9975	0.9862	LTR/Gypsy	LTR/Gypsy
NOMAD_I-int	1	0.9944	0.9962	0.986	LTR/Gypsy	LTR/Gypsy
Gypsy2_LTR	1	0.9927	0.9926	0.9802	LTR/Gypsy	LTR/Gypsy
STALKER4_I-int	1	0.9924	0.9899	0.977	LTR/Gypsy	LTR/Gypsy
Gypsy_LTR	1	0.9917	0.9893	0.9737	LTR/Gypsy	LTR/Gypsy
QUASIMODO_LTR	1	0.991	0.9858	0.9726	LTR/Gypsy	LTR/Gypsy
Chimpo_I-int	1	0.9908	0.9847	0.9672	LTR/Gypsy	LTR/Gypsy
DM176_I-int	1	0.9905	0.9773	0.9637	LTR/Gypsy	LTR/Gypsy
TIRANT_I-int	0.9996	0.9901	0.9767	0.9609	LTR/Gypsy	LTR/Gypsy
Gypsy1-I_DM	0.9995	0.9893	0.9737	0.9594	LTR/Gypsy	LTR/Gypsy
ROVER-I_DM	0.9995	0.9892	0.9697	0.9584	LTR/Gypsy	LTR/Gypsy
Chouto_I-int	0.9995	0.9884	0.9668	0.951	LTR/Gypsy	LTR/Gypsy
HMSBEAGLE_I-int	0.9994	0.9877	0.9648	0.9404	LTR/Gypsy	LTR/Gypsy
MDG1_I-int	0.9993	0.9862	0.9634	0.9371	LTR/Gypsy	LTR/Gypsy
Gypsy2-I_DM	0.9978	0.9847	0.9633	0.9323	LTR/Gypsy	LTR/Gypsy
Gypsy6_LTR	0.9975	0.9829	0.9611	0.9189	LTR/Gypsy	LTR/Gypsy
NOMAD_LTR	0.9972	0.9818	0.952	0.9189	LTR/Gypsy	LTR/Gypsy
Gypsy7_I-int	0.9971	0.9809	0.9481	0.9187	LTR/Gypsy	LTR/Gypsy
DM176_LTR	0.9967	0.9803	0.9458	0.9172	LTR/Gypsy	LTR/Gypsy
GTWIN_LTR	0.9967	0.977	0.944	0.8909	LTR/Gypsy	LTR/Gypsy
TABOR_I-int	0.9936	0.9763	0.9382	0.8883	LTR/Gypsy	LTR/Gypsy
ZAM_LTR	0.9924	0.976	0.9378	0.875	LTR/Gypsy	LTR/Gypsy
Gypsy3_LTR	0.9878	0.9758	0.9372	0.8618	LTR/Gypsy	LTR/Gypsy
Gypsy8_LTR	0.9856	0.9673	0.9313	0.8487	LTR/Gypsy	LTR/Gypsy
Invader5_LTR	0.9829	0.967	0.9266	0.8153	LTR/Gypsy	LTR/Gypsy
Invader6_LTR	0.9826	0.9594	0.9088	0.7913	LTR/Gypsy	LTR/Gypsy
Gypsy2_I-int	0.9803	0.9584	0.8928	0.7889	LTR/Gypsy	LTR/Gypsy
Stalker3_LTR	0.9799	0.958	0.8788	0.7823	LTR/Gypsy	LTR/Gypsy
Stalker2_I-int	0.978	0.9537	0.8774	0.7708	LTR/Gypsy	LTR/Gypsy
QUASIMODO2-I_DM	0.976	0.9433	0.8755	0.7659	LTR/Gypsy	LTR/Gypsy
Gypsy6_I-int	0.9737	0.936	0.8507	0.7531	LTR/Gypsy	LTR/Gypsy
Invader6_I-int	0.9673	0.9316	0.826	0.7468	LTR/Gypsy	LTR/Gypsy
QUASIMODO2-LTR_DM	0.9634	0.8933	0.8176	0.7249	LTR/Gypsy	LTR/Gypsy
Invader4_I-int	0.9634	0.8791	0.8153	0.7191	LTR/Gypsy	LTR/Gypsy
Gypsy10_I-int	0.9347	0.8683	0.8121	0.6838	LTR/Gypsy	LTR/Gypsy
TLD1	0.9226	0.867	0.8098	0.6429	LTR/Gypsy	LTR/Gypsy
DMTOM1_LTR	0.919	0.8586	0.7926	0.5415	LTR/Gypsy	LTR/Gypsy
Gypsy12_I-int	0.8953	0.8521	0.7868	0.5344	LTR/Gypsy	LTR/Gypsy
Gypsy12_LTR	0.8894	0.8468	0.7191	0.5176	LTR/Gypsy	LTR/Gypsy
GTWIN_I-int	0.8829	0.8233	0.7175	0.4756	LTR/Gypsy	LTR/Gypsy
ACCORD2_I-int	0.8742	0.8166	0.6856	0.4511	LTR/Gypsy	LTR/Gypsy
Gypsy5_I-int	0.8557	0.8097	0.6728	0.4214	LTR/Gypsy	LTR/Gypsy

Gypsy3_I-int	0.8421	0.7887	0.6429	0.3667	LTR/Gypsy	LTR/Gypsy
Gypsy8_I-int	0.7191	0.7147	0.5099	0.3133	LTR/Gypsy	LTR/Gypsy
Gypsy12A_LTR	0.596	0.6856	0.4439	0.3125	LTR/Gypsy	LTR/Gypsy
ZAM_I-int	0.582	0.5607	0.4362	0.2725	LTR/Gypsy	LTR/Gypsy
TV1I	0.5504	0.4371	0.4181	0.2238	LTR/Gypsy	LTR/Gypsy
TOM_I-int	0.4796	0.387	0.4018	0.1922	LTR/Gypsy	LTR/Gypsy
Gypsy11_LTR	0.3949	0.2536	0.293	0.1858	LTR/Gypsy	LTR/Gypsy
Ulysses_I-int	0.3594	0.1629	0.2797	0.1182	LTR/Gypsy	LTR/Gypsy
Gypsy9_I-int	0.2988	0.1304	0.2604	0.0817	LTR/Gypsy	LTR/Gypsy
Gypsy_DS	0.2882	0.108	0.1182	0.0405	LTR/Gypsy	LTR/Gypsy
Invader5_I-int	0.2436	0.0826	0.0725	0.0402	LTR/Gypsy	LTR/Gypsy
Gypsy11_I-int	0.0826	0.0471	0.0621	0	LTR/Gypsy	LTR/Gypsy
Gypsy9_LTR	0.0658	0	0.0457	0	LTR/Gypsy	LTR/Gypsy
OSVALDO_I-int	0.0389	0	0.0105	0	LTR/Gypsy	LTR/Gypsy
OSVALDO_LTR	0	0	0	0	LTR/Gypsy	LTR/Gypsy
TOM_LTR	0	0	0	0	LTR/Gypsy	LTR/Gypsy
TV1LTR	0	0	0	0	LTR/Gypsy	LTR/Gypsy
Ulysses_LTR	0	0	0	0	LTR/Gypsy	LTR/Gypsy
BEL_I-int	1	1	1	1	LTR/Pao	LTR/Pao
BEL_LTR	1	1	1	1	LTR/Pao	LTR/Pao
DIVER_I-int	1	1	1	1	LTR/Pao	LTR/Pao
DIVER_LTR	1	1	1	1	LTR/Pao	LTR/Pao
MAX_LTR	1	1	1	1	LTR/Pao	LTR/Pao
NINJA_LTR	1	1	1	1	LTR/Pao	LTR/Pao
ROO_I-int	1	1	1	0.9921	LTR/Pao	LTR/Pao
BATUMI_I-int	1	0.9993	1	0.9763	LTR/Pao	LTR/Pao
ROO_LTR	1	0.9968	1	0.9696	LTR/Pao	LTR/Pao
DIVER2_LTR	1	0.9923	1	0.961	LTR/Pao	LTR/Pao
ROOA_LTR	1	0.9866	0.9942	0.9275	LTR/Pao	LTR/Pao
MAX_I-int	1	0.9848	0.9886	0.9139	LTR/Pao	LTR/Pao
DIVER2_I-int	1	0.9816	0.985	0.9129	LTR/Pao	LTR/Pao
NINJA_I-int	0.9998	0.9745	0.9674	0.8445	LTR/Pao	LTR/Pao
ROOA_I-int	0.9974	0.9299	0.9591	0.7616	LTR/Pao	LTR/Pao
BATUMI_LTR	0.9949	0.9293	0.6912	0.7463	LTR/Pao	LTR/Pao
TRAM_I	0	0	0	0	LTR/Pao	LTR/Pao
TRAM_LTR	0	0	0	0	LTR/Pao	LTR/Pao
5S_DM	1	1	1	1	RNA	RNA
SSU-rRNA_Dme	1	0.9885	1	1	rRNA	RNA
LSU-rRNA_Dme	0.982	0.9846	1	0.9969	rRNA	RNA
LSU-rRNA_Hsa	0.0872	0.015	0.0089	0	rRNA	RNA
SSU-rRNA_Hsa	0.0354	0.0139	0	0	rRNA	RNA
ALUII_DM	1	1	1	1	Unknown	remaining
RSP	1	1	1	1	Satellite	remaining
XDMR	1	1	0.983	0.9834	Unknown	remaining
SAR2_DM	1	1	0.9542	0.8793	Satellite	remaining
NTS_DM	0.9333	1	0.7794	0.7708	Other	remaining

DMSAT6	0.8105	0.9	0.6194	0.7702	Satellite	remaining
SAR_DM	0.7202	0.7179	0.5484	0.4613	Satellite	remaining
ARS406_DM	0.6611	0.6289	0.5354	0.4561	Unknown	remaining
HETRP_DM	0.6393	0.534	0.5177	0.2548	Satellite	remaining
XDMR_DM	0.5871	0.4696	0.4001	0.2444	Unknown	remaining
IS3	0.4866	0	0.2283	0.0548	ARTEFACT	remaining
ALA_DM	0.2514	0	0.1263	0	Unknown	remaining
AT_rich	0.1599	0	0.0548	0	Low_complexity	remaining
AY1_DH	0	0	0	0	Unknown	remaining
DGRSAT1	0	0	0	0	Satellite	remaining
DGYSAT1	0	0	0	0	Satellite	remaining
DMR_DV	0	0	0	0	Unknown	remaining
DMRP1	0	0	0	0	Unknown	remaining
DMRPR	0	0	0	0	Unknown	remaining
DSAT	0	0	0	0	Satellite	remaining
DTSAT1	0	0	0	0	Satellite	remaining
FTZ_DM	0	0	0	0	Unknown	remaining
FUSHI_DM	0	0	0	0	Unknown	remaining
GC_rich	0	0	0	0	Low_complexity	remaining
IS1	0	0	0	0	ARTEFACT	remaining
IS10	0	0	0	0	ARTEFACT	remaining
IS150	0	0	0	0	ARTEFACT	remaining
IS186	0	0	0	0	ARTEFACT	remaining
IS2	0	0	0	0	ARTEFACT	remaining
IS3	0	0	0	0	ARTEFACT	remaining
IS5	0	0	0	0	ARTEFACT	remaining
LmeSINE1c	0	0	0	0	SINE/Deu	remaining
Tn1000	0	0	0	0	ARTEFACT	remaining

[a]A RepBase repeat can be represented by one or more consensuses of a particular RepARK library. A value of 1 means each position of the RepBase repeat is covered while 0 means that there is no evidence of this repeat in the corresponding RepARK library.

Supplementary Table 4. Repeat consensuses classified with TEclass.

	Library	Consensuses analysed[a]	DNA transposons	Retrotransposons	Not classified
**Sanger**	DmRepBase	249	20.5%	77.9%	1.6%
	ReASLib	391	25.8%	67.3%	6.9%
**simulated reads**	Repeat Scout	35,043[b]	53.7%	38.0%	8.3%
	wgs-assembler	14,147	43.7%	47.7%	8.6%
	RepARK CLC	1,239	38.6%	53.7%	7.7%
	RepARK Velvet	18,203	23.3%	69.4%	7.3%
**real reads**	Repeat Scout	11,439[c]	46.4%	46.3%	7.3%
	wgs-assembler	4,284	32.5%	60.8%	6.7%
	RepARK CLC	414	47.3%	44.2%	8.5%
	RepARK Velvet	14,296	35.9%	56.7%	7.4%

[a] Numbers of consensuses ≥50 bp in the respective libraries, which refers to all consensuses of DmRepBase, ReASLib, wgs-assembler, RepeatScout, simulated and real RepARK Velvet libraries. [b] 52% of consensuses in simulated RepARK CLC and 71% (3 Mb) of the overall length. [c] 53% of consensuses in real RepARK CLC and 80% (1.3 Mb) of the overall length.

Supplementary Table 5 Repeat annotation of the reference genome with TEclass annotated consensuses.

	Library	Consensuses analysed[a]	DNA transposons	Retro-transposons	Not classified	Total fraction
Sanger	DmRepBase	249	6.2%	18.0%	0.02%	24.2%
	ReASLib	391	5.9%	19.9%	0.9%	26.7%
simulated reads	Repeat Scout	1,239	4.3%	2.8%	0.6%	7.7%
	wgs-assembler	18,203	8.5%	18.2%	2.0%	28.7%
	RepARK CLC	35,043[b]	13.2%	17.3%	2.4%	32.9%
	RepARK Velvet	14,147	11.1%	17.7%	2.2%	31.0%
real reads	Repeat Scout	414	4.0%	0.7%	0.1%	4.8%
	wgs-assembler	14,296	4.1%	2.4%	0.4%	6.9%
	RepARK CLC	11,439[c]	12.1%	16.0%	1.6%	29.7%
	RepARK Velvet	4,284	8.1%	17.4%	1.9%	27.4%

[a] Numbers of consensuses ≥50 bp in the respective libraries, which refers to all consensuses of DmRepBase, ReASLib, wgs-assembler, RepeatScout, simulated and real RepARK Velvet libraries. [b] 52% of consensuses in simulated RepARK CLC and 71% (3 Mb) of the overall length. [c] 53% of consensuses in real RepARK CLC and 80% (1.3 Mb) of the overall length.

Supplementary Table 6: Completeness of each individual Alu element within the human RepARK *de novo* library.

name	completeness[a]		name	completeness[a]
AluYe2	0.2286		AluSq	0.8768
AluYe5	0.2883		AluSc	0.9107
AluYa4	0.3652		AluSq4	0.9158
AluYb3a2	0.3794		AluSx4	0.922
AluYf1	0.3936		AluSc5	0.925
AluYc5	0.4296		AluSg4	0.9647
AluYa5	0.5071		AluSg7	0.9681
AluYc2	0.5142		AluSx3	0.9681
AluYf5	0.5943		AluSp	1
AluYd3a1	0.5978		AluSq10	1
AluYbc3a	0.7562		AluSz	1
AluYa8	0.7722		AluSz6	1
AluYk12	0.805		AluJo	0.9894
AluYd8	0.863		AluJb	1
AluYi6	0.8975		AluJr	1
AluYk11	0.9255		AluJr4	1
AluYb9	0.9689		ALU	0.9263
AluYf2	0.9789		AluSg	0
AluYk13	0.9894		AluSx	0
AluYb3a1	0.9965		AluY	0
AluYg6	0.9965		AluYa1	0
AluYh9	1		AluYb8	0
AluSq2	0.5105		AluYc1	0
AluSc8	0.5957		AluYd2	0
AluSg1	0.6571		AluYd3	0
AluSx1	0.8339			

[a]An Alu element can be represented by one or more consensuses of the *de novo* library. A value of 1 means each position of the Alu repeat is covered while 0 means that there is no evidence of this repeat in the *de novo* library.
